# Serum thymidine kinase 1 protein concentration for predicting early progression and monitoring the response to TACE in hepatocellular carcinomas: a network meta-analysis

**DOI:** 10.2144/fsoa-2021-0016

**Published:** 2021-05-21

**Authors:** Hongbo Ma, Ailian Hei, Ji Zhou, Ellen He, Sven Skog, Jin Li

**Affiliations:** 1 Department of Medicine, Shenzhen SSTK Precision Medicine Institute, A301, Bulding 1, 2nd Yinxingzhijie, 1301-76 Guanguang Road, Longhua District, Shenzhen, China

**Keywords:** HCC, NMA, pre-HCC, STK1p, TK1, TACE

## Abstract

**Aim:**

A meta-analysis was conducted to evaluate the clinical significance of serum thymidine kinase 1 protein concentration (STK1p) in distinguishing between hepatocellular carcinomas (HCC**)** and non-HCC for predicting early progression and monitoring the response to transarterial chemoembolization in HCC.

**Materials & methods:**

A total of 24 eligible studies were included, containing 1849 HCC patients and 1069 healthy subjects.

**Results:**

The STK1p level significantly increased from normal controls to benign/pre-HCC and HCC (p < 0.0001). STK1p also increased significantly in sub-malignant groups: control being the lowest, followed consecutively by hepatic hemangioma, hepatitis B virus infection and hepatic cirrhosis (p < 0.05). After 1 month of transarterial chemoembolization treatment, STK1p level declined significantly, by 44.4% (p < 0.0001).

**Conclusion:**

STK1p is a useful prognostic biomarker in HCC.

## Lay abstract

There is a need for reliable prognostic biomarkers in liver cancer, to support therapy efforts. In this study we investigated if thymidine kinase 1 in blood could be used to discover patients with hepatocellular carcinoma and show the results of their treatment. The results are based on 1847 hepatocellular carcinoma patients and 1069 healthy people. We conclude that serum thymidine kinase 1 protein concentration is a reliable biomarker in liver cancer.

Hepatocellular carcinoma (HCC) is the second most common cause of death from cancer worldwide and occurs mostly in eastern Asia [1]. This rise in incidence is a result of liver cirrhosis, which is a common end point of various chronic liver diseases, such as hepatitis B (HBV) or hepatitis C (HCV) viral infections, alcoholic or nonalcoholic fatty liver disease (NAFLD), autoimmune hepatitis, biliary disorders and inherited metabolic defects [2,3], in addition to aflatoxin B1 (AFB1), major risk factors in China and India [4]. More than 60% of HCCs are found in developing countries, with approximately 50% of the cases in China are linked to HBV [2–4]. A standardized Liver Imaging Reporting and Data System for interpretation, reporting and data collection of imaging studies has been used for patients at the risk for developing HCC since 2008, improving the coherence between radiologists and clinicians and provide guidance for the management of HCC [5]. In Asia, where the prevalence of HCC is high, it is recommended to perform a combination of ultrasound and alpha-fetoprotein (AFP) measurements [6,7]. Additional biomarkers are also used, for example IL-6, GLDH, ALT, AST, GGT, alkaline phosphatase, total bilirubin and transferrin. These biomarkers are associated with higher risk of HCC, except for transferrin [8]. Combination of AFP, CA19-9 and carcinoembryonic antigen (CEA) does not provide a superior advantage over AFP alone. It is suggested that AFP is a screening and diagnostic tool for HCC detection [9]. However, since the diagnostic sensitivity of AFP is still around 60% and is fluctuating in chronic HBV or HCV patients, it may not be recommended as a surveillance test [5].

Cancer disease is a chronic proliferative disease. The process of precancerous disease from gene mutations in normal cell to abnormal cell proliferation will take a long time, around 10–30 years, before finally becoming a malignant tumor [10,11]. Moreover, AFP is not directly related to proliferating tumor cells [11–13]. Therefore, it is crucial to explore highly sensitive and specific proliferating serum biomarkers to achieve early discovery of invisible tumors in the pre-HCC process, offering a good chance of early treatment and cure.

Thymidine kinase 1 (TK1), a pyrimidine metabolic pathway enzyme involved in salvage DNA synthesis, is a cell cycle-dependent proliferation marker in human cells. The TK1 upregulation expression is highly dependent on the tumor cell growth [11,14–16]. Although the TK1 level in serum is low, reliable quantitation of serum thymidine kinase 1 protein concentration (STK1p) has been achieved by using a commercial enhanced chemiluminescence dot blot immunoassay (ECL-dot blot, SSTK Ltd., Shenzhen, China). Clinical evidence has supported that STK1p correlates significantly to tumor growth rates, clinical stages and evaluated for monitoring of tumor therapies and the prognosis of survival and relapse of various malignant tumors individually. Three network meta-analyses (NMAs) of lung (n = 2107) [17], colorectal (n = 1836) [18] and breast cancers (n = 1813) [19] conformed that the STK1p level increased significantly from normal to benign and then carcinomas (p < 0.0001). When the tumor was resected by extensive open surgery successfully, the level of STK1p decreased with a half-life of nearly 50%. In particular, a large data analysis of health screening (n = 160,086) demonstrated that STK1p was a reliable tool for assessment of early invisible/visible tumors [20]. In addition to STK1p, thymidine kinase activity also increased in solid malignancies. However, TK activity in serum is not specific for human tumors, since the TK1 enzyme in bacteria or viruses uses the same substrate as in human tumors. On the other hand, measuring STK1 by specific antibodies has been demonstrated to provide reliable results from human tumors [13,15]. So far, there are a number of publications using STK1p to monitor the treatment of HCC based on the ECL-dot blot. However, most of the publications are based on a limited number of cases, which may reduce the reliability of the conclusions.

In this study, we performed a NMA on 24 qualified studies according to the Preferred Reporting Items for Systematic Reviews and Meta-Analyses (PRISMA) guidelines. Our hypothesis is that STK1 is elevated in HCC patients and will decrease after transarterial chemoembolization (TACE) treatment. This is the first study evaluating the usefulness of STK1p for monitoring the treatment responses of TACE therapy in HCC and for identifying people with the risk to develop HCCs.

## Materials & methods

### Literature search

This retrospective NMA was performed according to PRISMA guidelines. The eligibility criteria included a thorough search of relevant articles from PubMed, EMBASE, the Cochrane Library, CNKI, Wanfang and VIP database until 30 April 2020 and was performed using the following medical subject headings: “thymidine kinase 1” or “serum thymidine kinase 1” or “TK1” or “STK1” or “STK1p”; “hepatocellular carcinoma” or “hepatoma” or “hepatic carcinoma”; “liver benign” or “hepatic benign” or “haemangioma”; “hepatic HBV” or “liver HBV”; “tumor” or “carcinoma” or “neoplasm” or “malignancy”; “hepatic cirrhosis” or “liver cirrhosis”. The search was restricted to human studies, but not to language.

### Inclusion criteria

The studies were reviewed, screened, and selected by two independent reviewers strictly following the inclusion and exclusion criteria. The inclusion criteria were: measuring the STK1p concentration; only using the enhanced chemiluminescence dot blot STK1p assay system from SSTK Ltd. with high sensitivity (77.9%) and specificity (99.7%) based on a specific anti-TK1 IgY antibody [11]; following international standard guidelines for diagnosing HCC; containing STK1p concentration data with mean and standard deviation, or data that could be re-estimated; including appropriate control groups such as tumor-free persons as controls, benign/pre-HCCs and HCCs before and after TACE treatment.

### Exclusion criteria

The exclusion criteria in this study were: insufficient data; using TK1 immunohistochemistry method or TK activity; studies containing unqualified data due to some technical faults; review articles and repeated literature; lack of appropriate control groups.

### Data extraction

The full text and the additional information for each study were carefully reviewed. After that, the following data were extracted from each study: first author’s name, publication year, the article title, publication journal, study population characteristics (design type, number of subjects, sources of control, gender, age) and relevant data for NMA (specimens, results). Two researchers independently assessed the quality of studies using the Newcastle-Ottawa Scale, and those with Newcastle-Ottawa Scale scores more than five points were included in the NMA. This NMA followed the PRISMA guidelines. Since all analyses were based upon previously published clinical studies, no ethical approval and patient consent were required.

### Statistical analysis

The NMA was performed with RevMan 5.1 software provided by Networks of Cochrane Review Groups and STATA 12.0 software (Stata Corporation, TX, USA). Initially, a heterogeneity test was conducted at the beginning. Then, according to the heterogeneity results, a fixed-effect model was selected when the I^2^ was lower than 50% and the p-value of the heterogeneity was >0.05; or a random-effect model was adopted when the I^2^ was greater than 50% and the p-value was <0.05 to calculate the weighted mean difference and 95% CI. Moreover, sensitivity analyses were performed with STATA version 12.0 software to evaluate the effects of excluding any individual study. Finally, Egger’s tests were conducted to examine publication bias. The p-value of STK1p between two different groups were also performed by Z test and p-values were calculated using RevMan 5.1 software. A p-value of <0.05 was considered to indicate significance.

## Results

### Literature search & study characteristics

**Figure 1.**
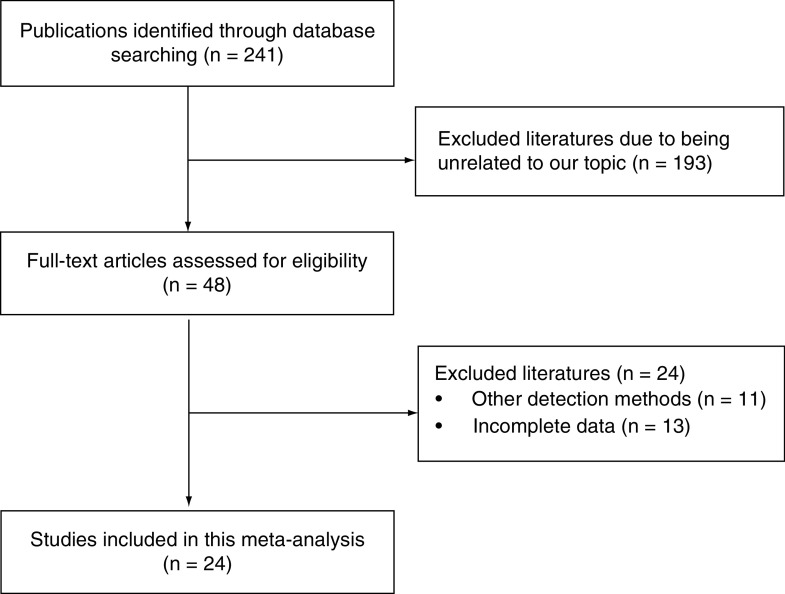
Flow chart of literature selection for the network meta-analysis.

Figure 1 illustrates the process of article retrieval and study selection. Initially, 241 publications were identified from various databases. After reviewing titles and abstracts, a total of 48 articles remained. Then, 24 of the remaining 48 articles were excluded since 11 articles used other STK1p detection methods which did not meet the inclusion requirements; 13 articles lacked complete data for conducting this network meta-analysis. As a result, 24 articles were included in the final analysis [21–44].

The main clinical features of the included studies were extracted and listed in Table 1. A total of 1847 HCCs, 1069 healthy subjects (volunteers) used as controls and 882 benign/pre-HCCs from the 24 studies between 2012 and 2019 were included in this NMA. All HCC cases in the study were confirmed by a pathological examination. In this study, about 68% (1121/1659) of HCCs were male. The age among the HCCs ranged approximately from 23 to 90. Among them, 407 HCCs were treated by TACE – a nonsurgical treatment for patients of intermediate stage (Barcelona Clinic Liver Cancer [BCLC] B) – and analyzed in respect to STK1p before and after TACE therapy. The time of TACE therapy was 4–6 weeks, depending the individual patient.

**Table 1.** Characteristics of the included literatures in the meta-analysis.

Study (year)	Tumor free (n)	Benign/pre-HCCs(n)	HCCs				TACE therapy	
			n	Male (n)	Female (n)	Age, mean (range)	Before	After
Ai M *et al.* (2012)	28		54	39	15	51 (45–63)	54	54
Ai M *et al.* (2015)	50		45	30	15	NA (35–79)	45	45
Chen ZR *et al.* (2019)	80		120	68	52	49.6 (34–66)		
Ding CZ *et al.* (2015)	80		80	48	32	63.99 (NA)		
Ding XL *et al.* (2019)		179	79			59.04 (37–80)		
Feng Q (2016)	32	42	76					
Gao FL *et al.* (2014)		13	100	78	22	NA (33–88)		
Gao FL *et al.* (2017)	50	50	104	71	33	58.67 (38–90)		
Hong YL (2013)	60		60	38	22	50.43 (44–68)	60	60
Jia SF *et al.* (2015)	40	40	40	20	20	42.93 (NA)		
Li J (2017)	55	55	85	56	29	52.6 (26–83)		
Li K *et al.* (2017)	30		33			53.6 (37–76)	33	33
Li ZP *et al.* (2016)	50	50	84	52	32	55.7 (34–65)		
Li ZY *et al.* (2018)^†^			45	30	15	58.8 (48–77)	45	45
			45	27	18	57.0 (45–79)	45	45
Pei QF *et al.* (2017)	80	80	88	51	37	56.8 (32–68)		
Qin JB *et al.* (2014)	63	85	112	72	40	54.5 (25–84)		
Tang YP *et al.* (2016)	40	30	125	107	18	NA (23–79)		
Wang F *et al.* (2017)	47	41	77	66	11	59.13 (46–79)		
Wang Q *et al.* (2013)	50	50	50	27	23	62 (NA)		
Wang SJ *et al.* (2018)			35	28	7	57.9 (35–80)	35	35
Xiao J *et al.* (2015)^†^			45	33	12	64.18 (NA)	45	45
			45	31	14	63.89 (NA)	45	45
Zhang SY *et al.* (2015)	104	109	116	75	41	57.8 (27–79)		
Zhao L *et al.* (2014)	50		48			NA (39–77)		
Zheng XW (2013)	80	58	56	45	11	NA (33–75)		
Total	1069	882	1847	1121	538		407	407

† In this Table 1, there were two separate patient groups which were treated with different types of chemotherapy. Each patient group included 45 cases.

HCC: Hepatocellular carcinoma; NA: Not available; TACE: Transarterial chemoembolization.

The normal controls of 1069 subjects (volunteers) in this NMA exclude those who had symptoms of tumors and/or infectious diseases but may include some diseases (invisible proliferating tumors/chronic inflammation/nontumor diseases, etc.). Therefore, the STK1p values were relatively higher than in those with normative STK1p data [45]. The normative STK1p values had excluded all diseases associated with tumor proliferation, in addition to all moderate/severe proliferating diseases, viral infectious, inflammation diseases, obesity, abnormal values of blood, urea or fecal tests, but could include some minor proliferating/chronic/nontumor diseases based on a study of 14,960 Chinese people (age: 20–79 years; male: 9586; female: 5374) of a health screening cohort of 42,383 persons [45], showing that the STK1p decreased slowly from 0.51 pmol/l (pM) at 20 years to 0.36 pM at 79 years, with a mean STK1p value of 0.35 pM. The results of normative STK1p values were similar to the disease-free STK1p (0.38 pM) in a routine health screening of 56,178 people [13].

Hepatic benign tumors mainly include hemangioma, focal nodular hyperplasia, perivascular epithelioid cell tumor and inflammatory pseudotumor [46].

Pre-HCC mainly include complications of liver cirrhosis (liver dysfunction, portal hypertension and the development of HCC), high risk infection of positive HBV and moderate to severe fatty liver, mainly including nonalcoholic steatohepatitis (NASH) and NAFLD [47].

### STK1p level between healthy subjects & HCCs

**Figure 2.**
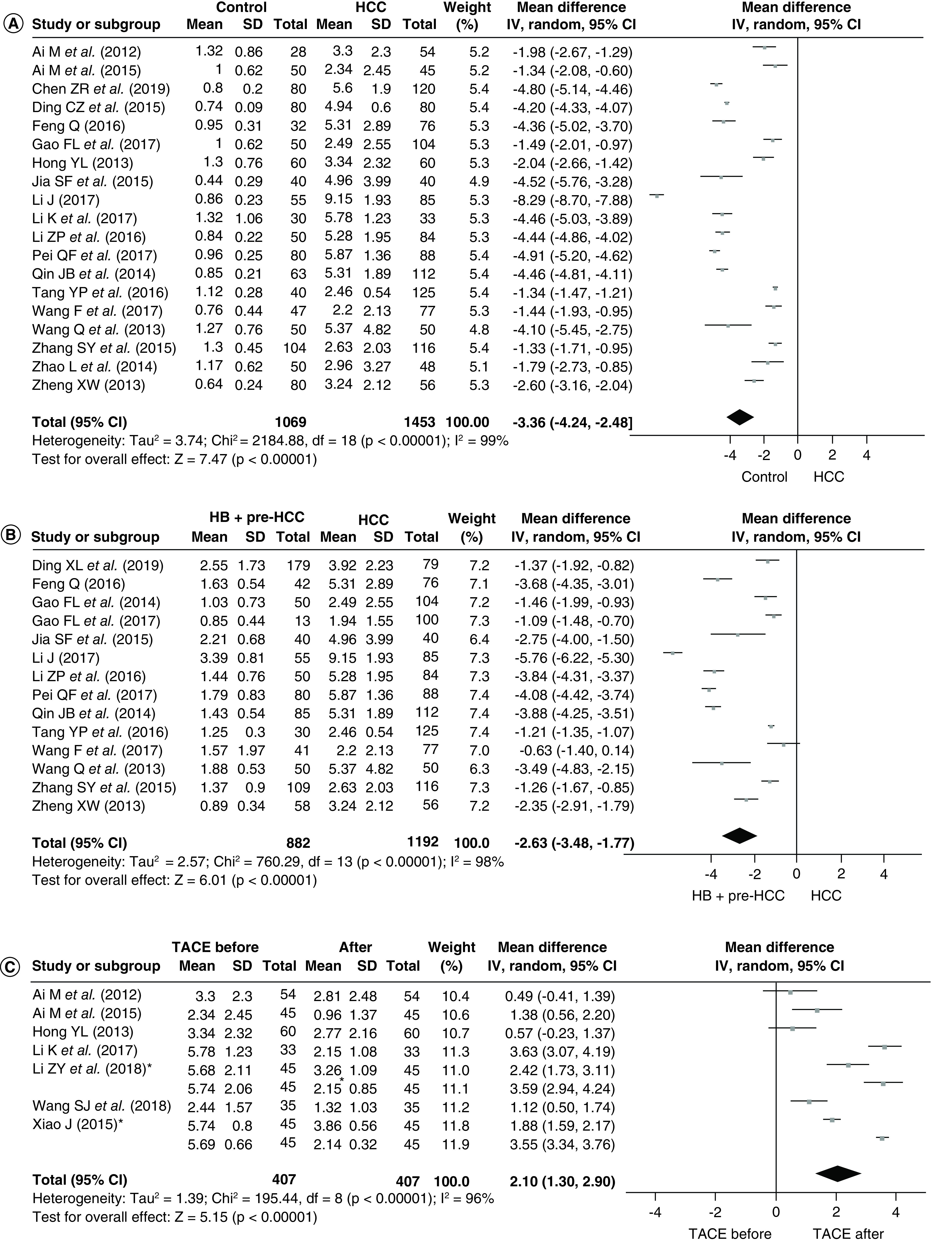
Forest plots of TK1. **(A)** Forest plots of healthy subjects and HCCs. **(B)** Forest plots of benign liver disease patients and HCCs. **(C)** Forest plots of HCCs before and one month after TACE. *In Table 1, there were two separate patient groups which were treated with different types of chemotherapy. Each patient group included 45 cases. Black diamond indicates the mean value. HCC: Hepatocellular carcinoma; IV: Inverse variance; SD: Standard deviation; TACE: Transarterial chemoembolization.

**Figure 3.**
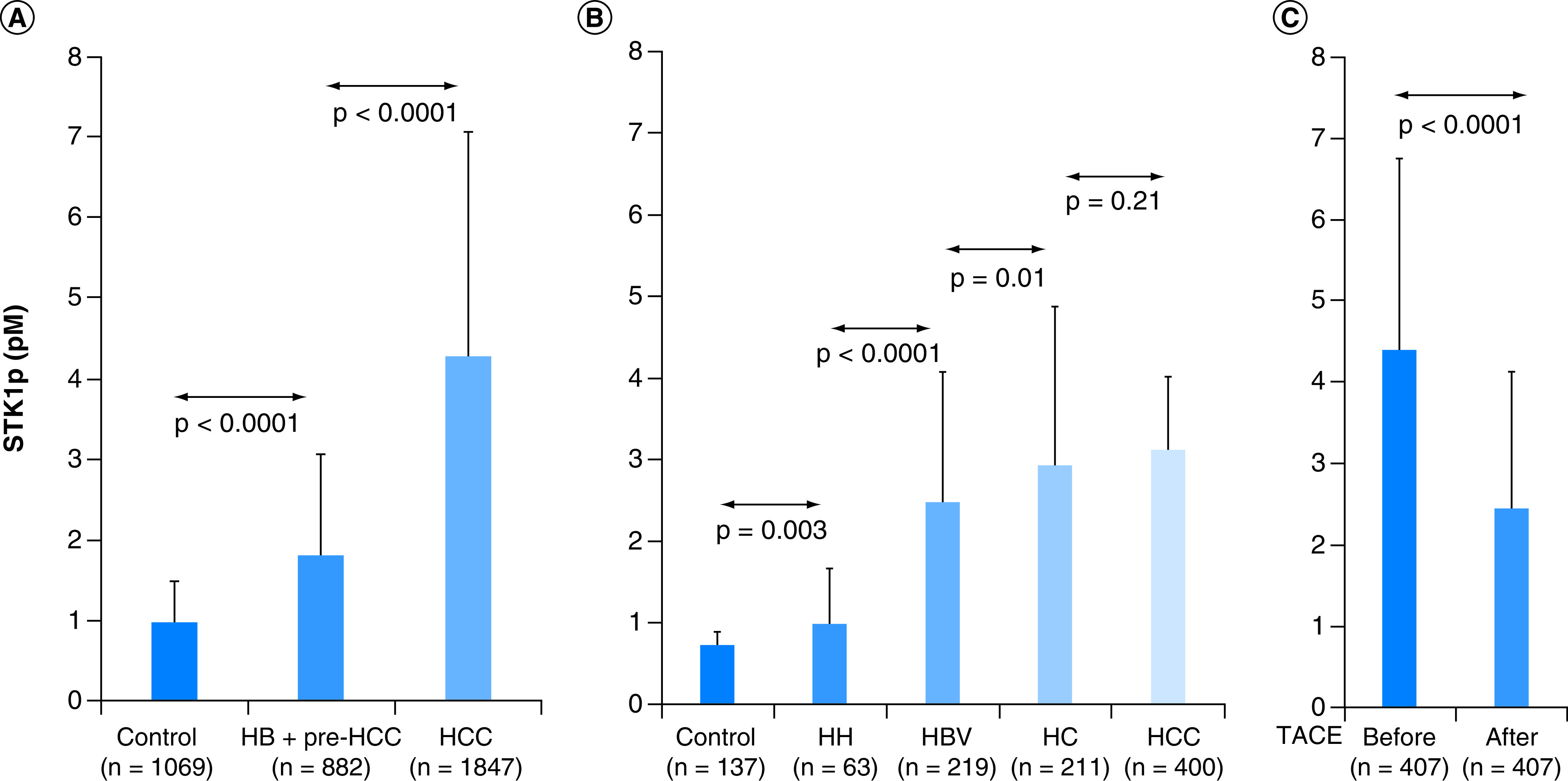
Concentration of TK1 in serum. Comparison of STK1p level among controls, hepatic benign/pre-HCC and HCC **(A)**; Comparison of STK1p level among controls, HH, HBV infections, HC and HCC **(B)**; Comparison of STK1p levels before and after TACE treatment in HCC **(C).** HBV: Hepatitis B virus; HC: Hepatic cirrhosis; HCC: Hepatocellular carcinoma; HH: Hepatic hemangiomas; STK1p: Serum thymidine kinase 1 protein concentration; TACE: Transarterial chemoembolization.

Of the 24 studies collected in this NMA, 19 were used for comparison of the STK1p values between tumor-free subjects and HCCs (Figure 2A & 3A). The number of the normal controls and HCC groups were 1069 and 1847, respectively. There were significant differences in the STK1p values between the two groups after the heterogeneity test (p < 0.00001; I^2^ = 99%). Therefore, a random-effect model was used to carry out the effect of the merging. The results showed that the weighted mean difference (MD) between the two groups was statistically significant at the 0.05 level (MD = -3.36; 95% CI [-4.24∼-2.48]; p < 0.00001). The level of STK1p among HCCs (4.29 ± 2.77 pM) was significantly higher than that among tumor-free subjects (0.97 ± 0.53 pM) (Figure 3A).

### STK1p level between hepatic benign + pre-HCCs & HCCs

Of the 24 studies used in this NMA, 14 were used to assess the difference in the STK1p values between benign liver disease patients with HCCs (Figure 2B). A total of 882 benign + pre-HCCs and 1192 HCCs were involved in this analysis, respectively. Significant differences were observed between the two groups after the heterogeneity test (p < 0.00001; I^2^ = 98%). Therefore, a random-effect model was used to analyze the effect of the merging. The results showed that a significant difference existed between the two groups (MD = -2.63; 95% CI [-3.48∼-1.77]; p < 0.00001).

### STK1p level in HCCs before & after TACE therapy

Of the 24 studies used in this NMA, the results of STK1p level in HCCs before and after TACE therapy were only from nine studies. We selected to evaluate the changes of STK1p concentration before and approximately 1 month after TACE therapy. The forest plots of the nine studies are shown in Figure 2C. Heterogeneity test results showed that the p-value was <0.00001 and I^2^ = 96%. Therefore, a random-effect model was adopted to examine the effect of the merging. The combined results indicated that statistical significance (MD = 2.10; 95% CI [1.30–2.90]; p < 0.00001) was found between the values before and around one month after TACE therapy groups.

### STK1p level between benign + pre-HCCs & HCCs

The STK1p level in HCCs (4.29 ± 2.77 pM) was significantly higher (p < 0.0001) than that in benign liver disease patients (1.81 ± 1.25 pM) (Figure 3A).

There were five publications among the 24 studies classified as hepatic hemangiomas (HHs), HBV infection and hepatic cirrhosis (HC) in relation to STK1p. We have summarized the data and show it in Figure 3B. The results show that the STK1p level correlates to the progression of the hepatic disease to HCC in the following manner: tumor-free is the lowest, followed by HH, HBV infection and HC (p < 0.05), but there was no significant difference between HC and HCC (p = 0.21).

The STK1p level after TACE therapy significantly declined (2.44 ± 1.68 pM) compared with the level before TACE therapy (4.39 ± 2.36 pM). The level of STK1p decreased by 44.4% after TACE therapy (p < 0.0001) (Figure 3C).

### Sensitivity analysis

**Figure 4.**
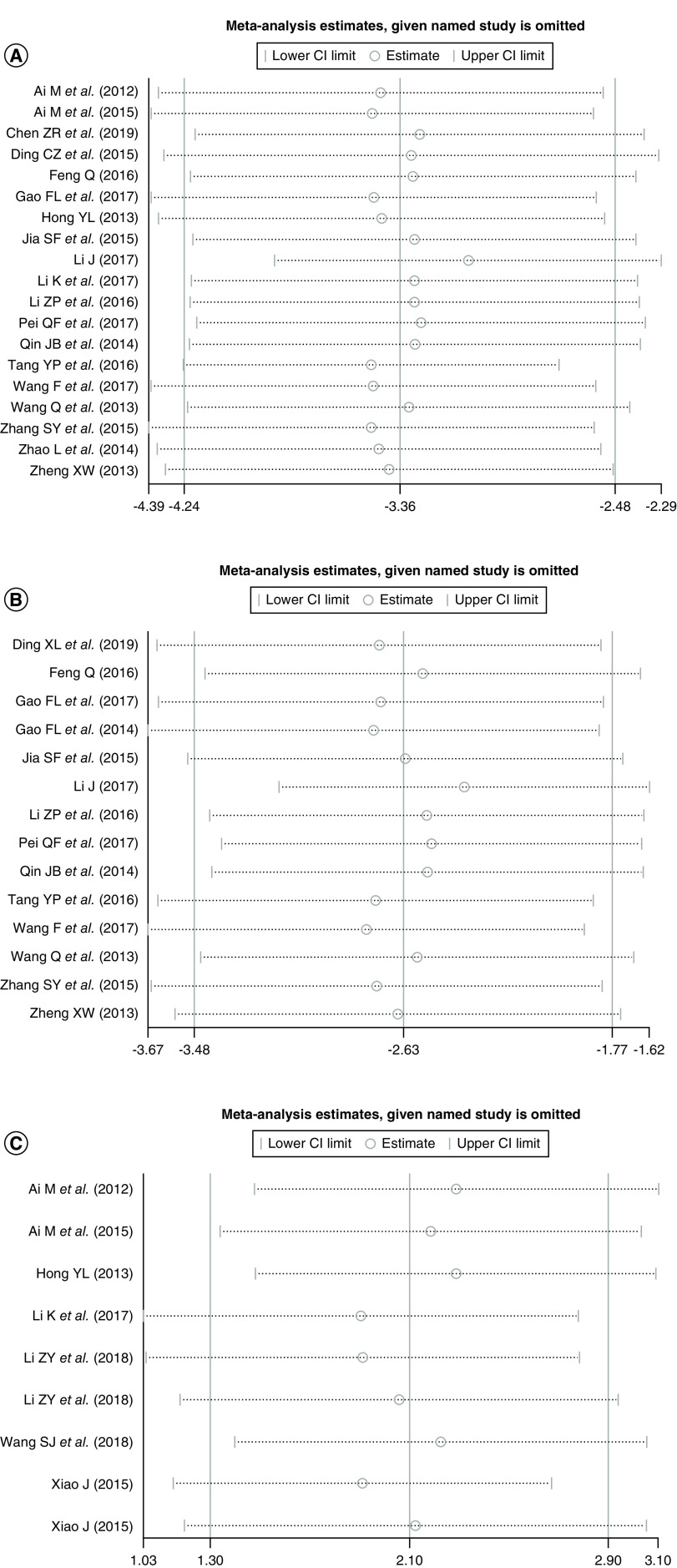
(A) Plots of the sensitivity between the controls and HCC group. **(B)** Plots of the sensitivity between benign + pre-HCC and HCC. **(C)** Plots of the sensitivity between before and after TACE treatment in HCC. HCC: Hepatocellular carcinoma; TACE: Transarterial chemoembolization.

Sensitivity analyses were conducted to evaluate the effects of excluding any individual study. Figure 4 presents the influence plots between the tumor-free and HCC (Figure 4A), between benign + pre-HCC and HCC groups (Figure 4B) and before TACE and after TACE groups (Figure 4C). By exclusion of one study at a time in turns, the results indicate that the sensitivity of the remaining studies did not substantially change in each comparison group.

### Publication bias

Egger’s linear regression tests were used to examine the potential publication bias. The results in Table 2 show that all p-values for the bias were >0.05, which demonstrates that no significant bias was identified among the publications in this net-work meta-analysis.

**Table 2.** Egger’s tests for the assessment of publication bias.

Standard effect	Coefficient	Standard error	T value	p > |t|	95% CI
Healthy vs HCC					
Slope	-2.821	0.693	-4.07	0.001	-4.283 to -1.359
Bias	-2.658	4.287	-0.62	0.544	-11.703 to 6.388
Benign vs HCC					
Slope	-1.186	0.678	-1.75	0.106	-2.663 to 0.291
Bias	-5.661	3.587	-1.58	0.140	-13.476 to 2.153
Before vs after treatment					
Slope	3.695	0.649	5.70	0.001	2.161 to 5.228
Bias	-5.130	2.953	-1.74	0.126	-12.113 to1.853

HCC: Hepatocellular carcinoma.

## Discussion

NMAs are the analysis of data from different studies in order to cover a larger number of cases [48]. A few things may limit our conclusions. In this retrospective NMA we strictly followed the rules for such analysis. According to the results shown in Figure 3B, the STK1p level increased significantly from the controls through benign/pre-HCC to HCC, and thus the STK1p was able to distinguish between these groups. This study also demonstrated high sensitivity, since the sensitivity did not change when individual studies were excluded. Moreover, the STK1p level (Figure 3C) declined significantly by about 45% after TACE treatment, corresponding to the half-life of the STK1p protein in serum. A similar half-life has been observed in other studies after extensive open surgery of about one month in patients with gastric [49], lung [17], colorectal and breast [19] carcinomas [18]. Thus, our NMA can be considered to be an objective and comprehensive summary of previous literature, making evaluation of new strategies more reliable. In respect to STK1p, the present NMA also supports how to use STK1p to evaluate early progression into pre-HCC, as well as monitoring the treatment effect of TACE.

Of the HCC staging systems in use today, BCLC is in favor when classifying HCC (stage 0, A, B, C and D). The early stages (BCLC 0/A) of HCCs are mostly treated with liver transplantation and other curative therapies. For the intermediate stage of HCC (BCLC B), locoregional treatments like TACE are mainly used [4]. Although TACE is an effective treatment for HCCs, potentially prolonging the survival time of those with HCCs by about 2 years, it is still far from satisfactory [50]. A more efficient therapeutic program should focus on discovery of pre-HCC stages as early as possible. It is also important to find effective serological biomarkers for monitoring treatment. STK1p – a tumor proliferating serological biomarker – is such a biomarker.

Prior studies proved that the ECL-dot blot assay of STK1p based on chicken IgY poly-antibody is a reliable technique for the early prediction of risk of premalignancies processing into malignancies in health screening [11,13,20]. This technique is also reliable for monitoring treatment of tumors and for assessment of survival and recurrence [11,20]. According to an investigation of a health screening study on 56,286 people, the cancer incidence rate increased by age from 0.032 in those of 21–40 years up to 0.44 above 60 years of age. A statistically significant increase of threefold–fivefold was also found in the cancer incidence rate between the elevated STK1p group and low STK1p group by progressive age (p < 0.00001) [11,51]. However, no significant correlations between STK1p and AFP, STK1p and CEA, or AFP and CEA were observed [13].

In this study, we found a significant close correlation between the STK1p values and sub-groups of hepatic disease (Figure 3B): normal controls had lower levels, followed consecutively by HH, HBV infection and HC (p < 0.05). However, there was no further increase in the STK1p values when HC developed into HCC, implying that the STK1p level in patients with HC had already reached a maximum value. STK1p did not further increase when those patients developed into malignancy. Similar results in a routine health screening of 56,178 subjects found that values of STK1p level in different liver diseases, including precarcinomas (moderate/severe fatty liver, HBV infection, obesity, HC, etc.) were increased significantly (p < 0.0001) in the following order: health-disease-free (0.38 pM), followed by HBV/obesity/moderate to severe fatty liver (0.54–0.69 pM) and HC (0.96), while AFP and CEA did not (p > 0.05) [13]. In a follow-up case study, a 72-year-old HBV-positive man was found to have liver pre-HCC in the right lower lobe by computed tomography, which was confirmed by pathological diagnosis. In the meantime, the STK1p value was elevated to 2.36 pM. The patient underwent surgery and chemotherapy, and 27 months later the patient was symptom-free, with a decrease in the STK1p value to 0.25–0.19 pM, while the AFP and CEA were still high (20). Thus, it is obvious that STK1p is more useful than AFP and CEA to distinguish pre-liver carcinoma groups.

Previous publications confirm that the early process of liver cancer development involves liver cirrhosis. Epidemiological studies show that the main cause of cirrhosis is viral infections (HBC, HBV), alcoholic (AFLD) or NAFLD, autoimmune hepatitis, biliary disorders and inherited metabolic defects (3), which finally end up as HCC. Thus, HCC development follows three steps: HBV, HCV, alcoholic/NAFLD; cirrhosis; HCC [50]. This means that the risk assessment of the premalignancy progress in liver cancer is an important point to investigate.

NASH is the progressive form of NAFLD, whose prevalence is steadily increasing because of the obesity epidemic resulting in complications of liver cirrhosis necessitating surgical resection in cases of diagnostic uncertainty and development of symptoms [7].

HH are the most common benign liver tumors, with an estimated prevalence of 0.4–20%. Most hemangiomas are small and generally grow. Routine follow-up by imaging is usually sufficient to avoid surgical intervention. Only the appearance of symptoms and enlarged hepatic hemangioma requires surgical intervention, most commonly by surgical methods, interventional radiology, TACE for giant hepatic hemangioma and emergency treatment for rupture of giant hepatic hemangioma [45,52]. The resection and hepatectomy are both safe and effective surgical treatments for hepatic hemangioma larger than 10 cm [53]. In this study, it was not possible to correlate the STK1p values to the size of HH, since the number of HH cases were too few. To confirm the STK1p correlation with size of HH for assessment of an effective surgical treatments, it is necessary to expand the number of cases. In general, the larger the benign tumor is, the greater the likelihood that it may developed into malignant cells in the further [10].

We concluded that the decrease in the STK1p value after TACE treatment reflects the treatment results. However, it is known that a side effect of TACE is deterioration of liver function, possibly due to unselective TACE destroying of normal liver tissue. However, we measure TK1 in serum (STK1p), not in liver tissue. In nonmalignant persons, very little TK1 is released from normal liver tissue into the blood/serum. This means that if the TACE treatment destroys normal liver cells it will not affect the TK1 level in the serum significantly.

In summary, STK1p measurement may have a valuable clinical relevance to distinguish between benign and different degrees of pre-HCC, and also evaluation of the effectiveness of the treatment of pre-HCC. Detection of liver tumor as early as possible and effectively differentiating between benign, pre-HCC and HCC will have great significance for formulating treatment plans and improving the survival rate. This is a large challenge in clinical work on hepatobiliary surgery and cure of patients.

Combining STK1p with other liver-associated biomarkers would be useful for HCC early risk detection of pre-HCC. For example, M30 antibody can detect fragments of keratins 18 (denoted as aKRT18) during apoptotic cell death and during the process of liver fibrosis in liver damage, and thus predict the severity of liver fibrosis and inflammation in chronic HBV patients [54]. M30 as a biomarker in combination with transient electrography resulted in the reliable identification in patients with an increased risk of progressed NAFLD [55–57], and was significantly elevated in patients with NASH compared with NAFL patients.

NMA is based on observations and therefore, the results may be affected by bias. The present NMA did not show any bias, according to the statistical analysis, but has some limitations [1]: there may still be some bias, in that the original research data of the specific pathological classification and staging of HCC may be not correct; [2] During assessing publication bias, the subjective influence of the researchers may affect the conclusion. However, regarding the limitations, the Egger’s test was performed to evaluate the effects of publication bias in order to minimize it [47].

Our study is a retrospective NMA, but it cannot replace a large randomized clinical trial, which should be done in the future. Although it takes a longer time and needs both human and material resources [58], NMAs based on individual patient data (network meta-analysis) are recommended for more reliable assessment of survival or treatment efficacy of patients. This will avoid the influence of bias. Recently, we performed a prospective individual patient data meta-analysis on non-small-cell lung carcinoma in relation to STK1p levels [59]. We conducted individual data from three hospitals and investigated the overall survival rate in patients. That was the first study using a real-world study approach, demonstrating that the STK1p was an independent prognostic factor for favorable overall-survival at clinical stages I–IIIA following a nonrandomized individual adapted treatment.

## Conclusion

Measuring STK1p level has clinical value in the screening of malignant cancers as an early warning indicator for HCC risk. STK1p may be a helpful approach for monitoring TACE treatment effectiveness and assessment of pre-HCC as early as possible. In addition, combining STK1p with other liver-associated biomarkers, for example, M30 may be useful for pre-HCC early risk detection. Future studies with a larger number of cases to validate these findings are necessary.

## Future perspective

More than 400 basic and clinical publications on TK1 have been published from more than 200 oncology clinics and health screening centers. The results show that TK1 is a useful prognostic biomarker for survival and recurrence, and for monitoring results of tumor therapy. These studies are based on an enhanced chemiluminescence dot blot STK1p assay system (SSTK Ltd.) using a specific chicken antihuman TK1 antibodies. The development of chicken TK1 antibodies increased the use of TK1 to almost all type of solid human tumors, and further promoted the interest of TK1 as tumor biomarker.

The antibody-TK1-methods we use today are time and resource consuming. However, we have developed a TK1-kit useful in automatic equipment that are now licensed for clinical use. To further reduce the resource, we are now looking to develop recombinant TK1 antibodies. These improvements together should reduce the analysis time to less than 2 h and the deviation (coefficient of variation [CV]) to less than 5%.

Although TK1 shows good correlations to clinical parameters, we recommend using TK1 with other tumor biomarkers depending on type of tumors studied.

**Summary points**

- Serum thymidine kinase 1 protein concentration has been used to discover patients with and without hepatocellular carcinoma (HCC) and the effect of treatment with transarterial chemoembolization.
- To improve the number of cases, we performed a network meta-analysis based on 24 clinical studies, including 1847 HCC patients and 1069 healthy people.
- The heterogeneity was high (p = 0.05) tested by a fixed-effect model.
- The sensitivity analyses were performed with STATA 12.0 software to evaluate the effects of excluding any individual study.
- No bias was found, tested by Egger’s analysis.
- Serum thymidine kinase 1 protein concentration was able to significantly distinguish between HCC and non-HCC and monitor the effect of the transarterial chemoembolization treatment.
